# Pathoadaptive Mutations in *Salmonella enterica* Isolated after Serial Passage in Mice

**DOI:** 10.1371/journal.pone.0070147

**Published:** 2013-07-25

**Authors:** Sanna Koskiniemi, Henry S. Gibbons, Linus Sandegren, Naeem Anwar, Gary Ouellette, Stacey Broomall, Mark Karavis, Paul McGregor, Alvin Liem, Ed Fochler, Lauren McNew, Carolyn Nicole Rosenzweig, Mikael Rhen, Evan W. Skowronski, Dan I. Andersson

**Affiliations:** 1 Department of Medical Biochemistry and Microbiology, Uppsala University, Uppsala, Sweden; 2 US Army Edgewood Chemical Biological Center, Aberdeen Proving Ground, Aberdeen, Maryland, United States of America; 3 Department of Microbiology, Tumor and Cell Biology, Karolinska Institute, Solna, Sweden; 4 Science Applications International Corporation, Aberdeen Proving Ground, Aberdeen, Maryland, United States of America; 5 OptiMetrics, Inc., Abingdon, Maryland, United States of America; Indian Institute of Science, India

## Abstract

How pathogenic bacteria adapt and evolve in the complex and variable environment of the host remains a largely unresolved question. Here we have used whole genome sequencing of *Salmonella enterica* serovar Typhimurium LT2 populations serially passaged in mice to identify mutations that adapt bacteria to systemic growth in mice. We found unique pathoadaptive mutations in two global regulators, *phoQ* and *stpA*, which increase the competitive indexes of the bacteria 3- to 5-fold. Also, all mouse-adapted lineages had changed the orientation of the *hin* invertable element, resulting in production of a FliC type of flagellum. Competition experiments in mice with locked flagellum mutants showed that strains expressing the FliC type of flagellum had a 5-fold increase in competitive index as compared to those expressing FljB type flagellum. Combination of the flagellum cassette inversion with the *stpA* mutation increased competitive indexes up to 20-fold. These experiments show that Salmonella can rapidly adapt to a mouse environment by acquiring a few mutations of moderate individual effect that when combined confer substantial increases in growth.

## Introduction

Bacteria adapt genetically to changing environments and many studies show that bacteria possess a considerable potential to increase fitness during experimental evolution under constant laboratory conditions [1], [2]. However, less is known about how bacteria adapt to a more complex and variable environment, such as that encountered inside a host. *Salmonella enterica* serovar Typhimurium (hereafter referred to as *S. typhimurium*) is a common facultative intracellular pathogen that can adhere to and invade gut epithelial cells. After invasion, bacteria that reach the submucosa can be internalized by macrophages and disseminate to systemic sites such as the lymph nodes and ultimately the spleen and liver [3]. To be able to survive and replicate in these various growth niches, *S. typhimurium* possesses an arsenal of virulence factors which, when properly regulated, provide an appropriate physiological response to the actual environment [4] In addition, upon growth inside a host, selection can benefit bacterial mutants with altered expression of virulence genes that better suit the environment of the host. These mutations can be considered pathoadaptive mutations. Most studies of virulence genes use mutants where potential genes involved in virulence are inactivated or altered, to confirm loss/increase of virulence [5], [6], [7] This does not however give any information to what actually happens during growth and adaptation in natural settings. Another approach to study pathoadaptive mutations is by comparative genomic or proteomic analyses, where the genomes of pathogenic bacteria can be compared to those that are avirulent to determine what changes might be responsible for that change [8], [9]. However, these studies rarely identify single mutations responsible for the increased virulence, which makes it hard to elucidate the exact gain of each mutation.

Here we study pathoadaptive mutations in *S. typhimurium* found after serial passage of bacteria in mice followed by whole genome sequencing of the adapted populations. Previous results from our laboratory show that *S. typhimurium* evolved experimentally by serial passage for <200 generations of growth in mice rapidly increased their growth rate as measured by an up to 50-fold increase in competitive index [10] Here we identify the mutations responsible for faster growth in mice using whole genome sequencing and verify their role in pathoadaptation by reconstructing the mutations in wild-type backgrounds. In recent years, microbial whole-genome sequencing has advanced from identifying the genomes of model-organisms, to be used as a tool in determining adaptive mutations and comparative analysis of pathogenic bacteria. This type of whole-genome sequencing has applications to the fields of bacterial pathogenesis and vaccine development [11] epidemiology [12] and microbial forensics [13], [14].

We find unique mutations in two global regulators of virulence associated genes, *phoQ* and *stpA*, that increase the expression of the *spv* (*s*almonella *p*lasmid *v*irulence) locus required for survival within macrophages. We also observed fixation of bacteria expressing one type of flagellum (FliC) rather than the other (FljB). Finally, we show that even though any of the mutations alone only give a relatively small increase in growth rates in mice (3- to 5-fold increase in competitive index (CI)), the combined effect of the mutations can result in a considerable increase in CI.

## Materials and Methods

### Preparation of Isolates/DNA for Sequencing

DNA was prepared from populations of cells after extraction from mice spleens and after few generations of growth in Luria Bertani (LB) broth. DNA was prepared with Qiagen genomic tip 500G kit (Qiagen) according to the manufacturer and tested for concentration and purity with a Nanodrop 1000 (Thermo Scientific). Only DNA with A_260_/A_280_ ratios larger than 1.8 were used for sequencing.

### Whole Genome Sequencing

Shotgun libraries were prepared from genomic DNA isolated from the JB124 parent strain and from populations derived from each host-adapted lineage. DNA was sheared by nebulization and converted into shotgun libraries according to standard protocols for the Roche/454 procedure [15] Shotgun sequencing was performed on the Roche/454 GS-FLX instrument using the Titanium reagent package according to manufacturer’s protocols. Mean coverage for all isolates was between 20 and 35-fold. Datasets for each lineage have been deposited in the NCBI Short Read Archive (SRA) under the project submission ID SRA075312 with SRA accession numbers SRS418168-SRS418176.

### Bioinformatic Analysis

Reads were assembled *de novo* using Newbler GSAssembler (version 2.3) to confirm the quality of raw sequence data. Reads from all strains were mapped to the published *S. typhimurium* LT2 reference sequence (NC_003197.1 and NC_003277.1 for the chromosome and pSLT1 virulence plasmid, respectively) using both GSMapper and CLC Genomic workbench v 5.1 (CLC Bio, Aarhus Denmark). Mutations present in the JB124 strain the LT2 reference strains were first identified to focus the search for adaptive mutations on those mutations that were unique to the adapted strains and absent from the parent strain. High-confidence differences (HCDiffs) were identified based on the neighborhood quality score algorithm in Newbler and the SNP and DIP mapping algorithms in CLC Genomic Workbench. SNPs and small insertion/deletion events in strain JB124 relative to the LT2 reference sequence were initially identified based on an 85% read-difference cut-off. In addition, structural variations called by GSMapper were identified as regions that exhibited rearrangements (deletions or inversions) with obvious and consistent join points. The latter class of variations presents as a collection of reads that map consistently to two places on the genome with common break points. All mutation calls and putative rearrangements were verified by manual inspection of the assembly files and by PCR analysis. The candidate mutations identified in JB124 were compared to all published *Salmonella* genomes to help screen out sequencing errors in the published sequence. Residual sequencing errors in the LT2 genome were identified by comparing the mutations identified by 454 sequencing of JB124 with differences between LT2 and 14028s using MAUVE [16]. The presence and relative amount of identified mutations was verified with colony PCR and subsequent sequencing with StpA and PhoQ primers.

### Genetic Reconstruction of Adaptive Mutations

Possible pathoadaptive mutations identified were reconstructed in wild type background to analyze their contribution to the increased growth rates in mice. Resistance cassettes (*cat*) were inserted ∼5 kb from the identified mutation in genes with no known function (STM2804 and STM1236) with linear transformation as described previously [17]. In short, linear DNA was amplified using STM2804_cat_ins and STM1236_cat_ins primers with Phusion enzyme according to the manufacturer (Finnzymes) according to the following protocol in a Geneamp 9700 (Applied biosystems); 94°C 2 min, then 31 rounds of 94°C 30 s, annealing (68–72°C) 30 s, elongation 72°C (30 s-2 min) and a final elongation at 72°C for 7 min before cooling down to 4°C. As template for the *cat* gene the pKD3 plasmid prepared with E.Z.N.A plasmid mini kit (Omega-Bio-Tek) was used. The resistance marker from pKD3 inserted by linear transformation included FRT-recombination sites present on the template plasmids. PCR products of the right size were purified with Fermentas gel and PCR extraction kit (Fermentas) according to the instructions of the manufacturer and used for linear transformation as described previously [17]. Resistance cassettes were transferred to strains with mutations using P22 transductions [18]. The presence of the mutations were verified with PCR and subsequent sequencing before a new P22-lysate was made on strains now carrying a *cat*-resistance marker ∼5 kb from the mutation. The mutation was transferred to fresh wild type background with and without a selectively neutral *cobI*-24::MudJ mutation, using P22 transduction with the prepared lysates and isogenic strains carrying the mutation (wt) or not (*cobI*-24::MudJ) were saved for the mice competition experiments. Resistance markers were removed from strains using plasmid pCP20 carrying the FLP-recombinase under thermal induction control [17] for the flagellum *fljBA* ^ON^/*fljBA* ^OFF^ mutants. The original *fljBA* ^ON^/*fljBA* ^OFF^ mutants were a gift from Kelly T. Hughes [19] and the mutations were transferred into our mutant and wild type backgrounds by P22 transductions. Presence of the *fljBA* ^ON^/*fljBA* ^OFF^ mutations was verified with PCR and restriction cleavage with *Sac*I, which cleaves the *fljB* ^OFF^ PCR product (to 419 bp +1329 bp) but not the *fljB* ^ON^ product (1748 bp). Presence of the identified mutations was verified after *fljBA* ^ON^/*fljBA* ^OFF^ transductions with PCR and sequencing.

### Competition Experiments in Mice

The competition experiments were performed in female 6–8 weeks old BALB/c mice. The mutants were competed against respective isogenic wild type strain (Table 1) in the following pairs DA18243/DA18336, DA18251/DA18344, DA18933/DA18938, DA18935/DA18938, DA18939/DA18942, DA18940/DA18942, DA21181/DA21184, DA21182/DA21184 and DA21179/DA21185. Five mice/group were infected intraperitoneally with a mixture of mutant and wild type bacteria at a ratio of 1∶1 and a final amount of ∼10^4^ cfu. Mice were sacrificed on day 3–4 and the spleens from sacrificed mice were removed and homogenized in PBS before dilutions of the homogenates were plated on LA and LA plates supplemented with Kanamycin. The competition index was calculated after one cycle of growth in mice (∼10 generations) as the ratio of the mutant population divided by the population of the isogenic wild type strain as compared to the proportion of the mutant population relative to the wild type in the infection mixture. Selection coefficients (*s*) were estimated using the regression model s = [ln(R(t)/R(0))]/[t], as previously described [20], where R is the ratio of mutant to wild type and t the number of generations. The relative fitness (W) was calculated as 1-*s.*

**Table 1:** Strains of *S. typhimurium* used in this study.

Designation	Relevant genotype	Origin
JB124	Wild type LT2	Lab collection^10^
DA5803	Mouse evolved lineage 1	Lab collection^10^
DA5810	Mouse evolved lineage 2	Lab collection^10^
DA5816	Mouse evolved lineage 3	Lab collection^10^
DA5822	Mouse evolved lineage 4	Lab collection^10^
DA5828	Mouse evolved lineage 5	Lab collection^10^
DA5884	Mouse evolved lineage 6	Lab collection^10^
DA5894	Mouse evolved lineage 7	Lab collection^10^
DA5815	Mouse evolved lineage 8	Lab collection^10^
DA18243	*stpA*[CTT124Δ] (E42Δ), STM2804::*cat*	This work
DA18251	*phoQ*[A38T] (L13Q), STM1236::*cat*	This work
DA18336	STM2804::*cat, cobI-*24::MudJ(KanR)	This work
DA18344	STM1236::*cat, cobI-*24::MudJ(KanR)	This work
DA18933	*stpA*[CTT124Δ] (E42Δ), STM2804::FRTscar, Δ*hin*5717:FRT*cat*FRT (*fljBA*OFF)	This work
DA18935	*stpA*[CTT124Δ] (E42Δ), STM2804::FRTscar, Δ*hin*5718:FRT*cat*FRT (*fljBA*ON)	This work
DA18938	ST2804::FRTscar, *cobI-*24::MudJ(KanR), Δ*hin*5718:FRT*cat*FRT (*fljBA*ON)	This work
DA18939	*phoQ*[A38T] (L13Q), STM1236::FRTscar, Δ*hin*5717:FRT*cat*FRT (*fljBA*OFF)	This work
DA18940	*phoQ*[A38T] (L13Q), STM1236::FRTscar, Δ*hin*5718:FRT*cat*FRT (*fljBA*ON)	This work
DA18942	STM1236::*cat, cobI-*24::MudJ(KanR), Δ*hin*5718:FRT*cat*FRT (*fljBA*ON)	This work
DA21179	Δ*hin*5717:FRT*cat*FRT (*fljBA*OFF)	This work
DA21185	*cobI-*24::MudJ(KanR), Δ*hin*5718:FRT*cat*FRT (*fljBA*ON)	This work

### Ethics Statement

Animal experiments were carried out at the animal facility at the Microbiology and Tumor Biology Centre, Karolinska Institute (Stockholm, Sweden), in compliance with EU, national and institutional guidelines (regulations DFS2004:4 and DFS2004:15 from Swedish Board of Agriculture). Animal experiments were approved by the Animal Experiments Committee, Stockholm North (ethical permits N345/08 and N491/11).

### Quantitative Real-time PCR

Cells were grown overnight and diluted 1∶100 in fresh LB and grown to OD_600_ = 0.5. 1 ml of the culture was removed and RNA was prepared with the SV Total RNA Isolation System (Promega) according to the manufacturer. RNA was treated with turbo DNAse (Ambion) according to the instructions of the manufacturer to remove any remaining chromosomal DNA. RNA concentrations were measured with a Nanodrop 1000 (Thermo scientific) and 0.1- to 0.3 µg RNA was used for cDNA synthesis. mRNA was converted to cDNA using the iScript reverse transcription kit from BioRad according to the manufacturer. Quantitative real-time PCR technique based on the high affinity of SYBR Green dye for double-stranded DNA was used to measure relative mRNA levels with the CT-value method according to the manufacturer (Bio-Rad). The fluorescence signal was monitored on-line, using the iCycler real-time PCR system (Bio-Rad laboratories). The mRNA levels were calculated relative to *dnaE* mRNA in each individual RNA-sample, and normalized to expression of the same gene in wild type cells (DA6192). Primers for the real-time PCR are found in Table S4. RNA was prepared from 2 independent cultures and all quantitative real time PCR measurements were run in duplicates and at least 2 times for all samples.

### Motility Testing

To test whether the motility of cells expressing *fliC* type of flagellum rather than *fljB* type flagellum was altered, we performed motility testing on motility agar plates as described previously [21]. Motility plates were made with 0.3% agar, 0.5% Tryptone, 0.25% Yeast extract and 0.25% Sodium chloride. In short, 5 fresh colonies (after growth over night on plate) were stabbed into fresh (less than 24 h old) motility agar plates with a toothpick. Motility was detected visually after 16 h of growth in 37°C as the spreading of the colony in the motility agar. Motility was given as a five grade scale; −, (+), +,++ and +++, where − stands for non-motile and +++ for fully motile.

## Results

### Serial Passage of Bacteria in Mice

**Figure 1:**
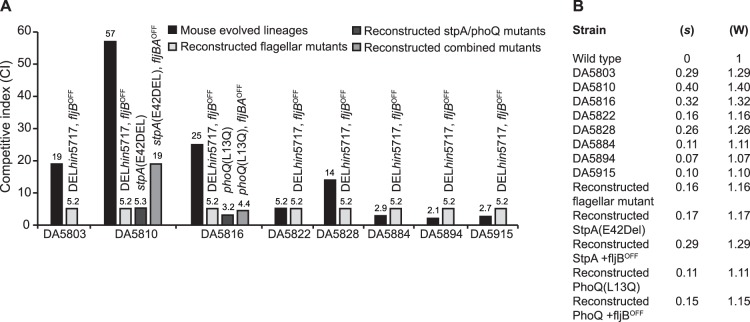
Competitive indexes of reconstructed and parental mutants.

In a previous study, 8 independent lineages of *S. typhimurium* were evolved by serial passage in BALB/c mice for approximately 130 generations [10]. All of these evolved bacteria (listed in Table 1) showed an increased growth rate in mice as measured by competition with the non-evolved parent strain. The competition indexes (CI) were calculated as the ratio of the evolved population per parent after 10 generations of growth in mice and varied between 2 and 57 for the different lineages (Fig. 1a). This corresponds to an increase in relative fitness varying from 10- to 40% for the different evolved populations (Fig. 1b).

### Whole-genome Sequencing

The eight evolved lineages and their wild type ancestor JB124 were sequenced to between 25- and 42-fold mean sequence coverage. Based on the overall similarity in quality of each of the 9 datasets, we deemed it likely that the results of comparison of each dataset to the published reference sequence would be valid (Table S1). We identified 89 High-confidence differences (HCDiffs) between JB124 and the published sequence of *S. typhimurium* LT2 (Genbank ID NC003197) (Table S2). These differences can either be the result of sequencing errors in the published reference strains or accumulation of mutations since the separation of these isolates. Considering the number of changes observed we find the former explanation more likely. Screening of the HCDiffs against other complete *S. typhimurium* genomes in the non-redundant databases also support this and we find only 13 mutations that appear to be unique to JB124 (Table S3). Almost all of these mutations were also identified in adapted strains. Although coverage of the reference genome was consistently above 99% some regions of JB124 sequenced poorly and had some remaining apparent gaps. No lineage-specific mutations that might be candidates for pathoadaptive mutations were observed in those regions.

### Identification of Pathoadaptive Mutations

After screening out differences between the published reference and the parent strain, we looked for both nucleotide substitutions and genomic rearrangements between JB124 and the evolved lineages. Each of the lineages examined contained at least one of each type of variation as described below. In typical sequencing projects, mutations in a sample derived from a pure, single-colony purified clone are identified on the basis of high consensus disagreement with the published reference sequence (>85% read-level consensus for all mutations; 100% for most). However, since our sequencing data for the adapted strains was derived from populations obtained from mouse serial passage instead of single clones, only mutations fixed in the population would meet the 85% consensus cut-off. No such high frequency variations were detected. With a lower cut-off criterion we identified 12 different high-confidence mutations that were present in 11- to 60% of the reads (Table 2). These mutations were lineage-specific, suggesting that they had been selected independently during repeated animal passage and possibly conferred an increased competitive ability in mice. Mutations mapping to homopolymer regions known to present problems in 454 sequences were ignored, as were candidate mutations mapping close to the ends of contigs, which can present assembly conflicts. The identified mutations were mostly substitutions, as well as few insertion/deletions. Among these 12 mutations, we chose to study further the mutations that had reached the highest level in the population, and that were expected to confer the most beneficial effect. Accordingly, the populations designated DA5810 and DA5816 contained point mutations in the *stpA* (60% of the population) and *phoQ* (57% of the population) genes (positions 2947501 and 1318652) respectively. Both of these genes are known regulators of virulence factors in *Salmonella* and were thus examined further.

**Table 2:** High-confidence candidates for pathoadaptive mutations.

	Lineage	Pos Start	Pos End	Ref	Variant	# Reads	% Different	Gene affected	AA sequence^a^	Function
**Chromosome**	DA5822	328501	328501	A	–	21	52%	STM0286	Frameshift	Putative cytoplasmic protein, mutation fuses STM0286 to STM0287 (putative periplasmic protein)
	DA5803 DA5816	392852	392852	A	G	28	11%	STM0350.S	L342P (455)	Outer membrane efflux-like protein^b^
	DA5803	1099934	1099934	C	T	18	22%	STM1007	I54V (79)	Hypothetical protein
	**DA5816**	**1318652**	**1318652**	**A**	**T**	**21**	**57%**	***phoQ***	**L13Q (487)**	**Transmembrane sensor kinase, regulates resonse to low Mg^2+^ and cationic peptides**
	DA5894	1852977	1852977	T	C	13	31%			Intergenic
	DA5915	2242837	2242837	C	A	28	11%	*stcD*	D335Y (335)	Putative outer membrane lipoprotein
	DA5915	2942948	2942948	T	C	24	37%			Intergenic, between *gapP* and *gabT*
	**DA5810**	**2947501**	**2947503**	**TTC**	**–**	**25**	**60%**	***stpA***	**ΔE42 (42/134)**	**DNA binding protein, nucleoid associated. Represses RpoS regulon, homolog of H-NS**
	DA5822	3804942	3804942	C	A	37	30%	*yhjS*	Truncation (93/523)	*bcsE* homolog, required for cellulose biofilm synthesis
	DA5822	3807045	3807045	G	A	32	22%	*yhjU*	Truncation (209/559)	Putative cellulose biosynthesis protein
	DA5816	4402521	4402521	C	T	22	14%	*aceB*	Synonymous	Malate synthase
**pSLT**	DA5803 DA5816	35606	35606	A	G	47	15%	*rlgA*	M>T (391/554)	Putative integrase protein

a Reference>Variant (position in protein/total protein length).

b Variant also found in DA5822 but not called as high-confidence (3 non-duplicate reads).

We confirmed the presence of the *stpA*(E42delta) and *phoQ*(L13Q) alleles by sequencing PCR products from 10 single colonies of the populations. This analysis confirmed that both mutations were present in 50–60% of their respective population (6/10 and 5/10 respectively). StpA is a H-NS homologue and has no assigned function in *Escherichia coli* (*E. coli*) [22], [23]. However, in *S. typhimurium* StpA was recently shown to regulate expression of 5% of the genes in the *Salmonella* genome [24]. An important role of StpA in *S. typhimurium* is growth phase-specific regulation of gene expression. For example, during mid-exponential phase StpA prevents expression of the RpoS regulon whereas it is required for full expression of the CRP-cAMP regulon during late-exponential growth [24]. PhoQ is the sensory componant in the PhoQ/PhoP two-component regulatory system [25]. Inactivation of either *phoP* or *phoQ* has been shown to attenuate virulence of *S. typhimurium* in mice by compromising survival in mice macrophages [5], [26]. Thus, it is unlikely that the *phoQ* (L13Q) mutation found here is a loss of function mutation.

### Alignment of PhoQ and StpA Protein Sequences

**Figure 2:**
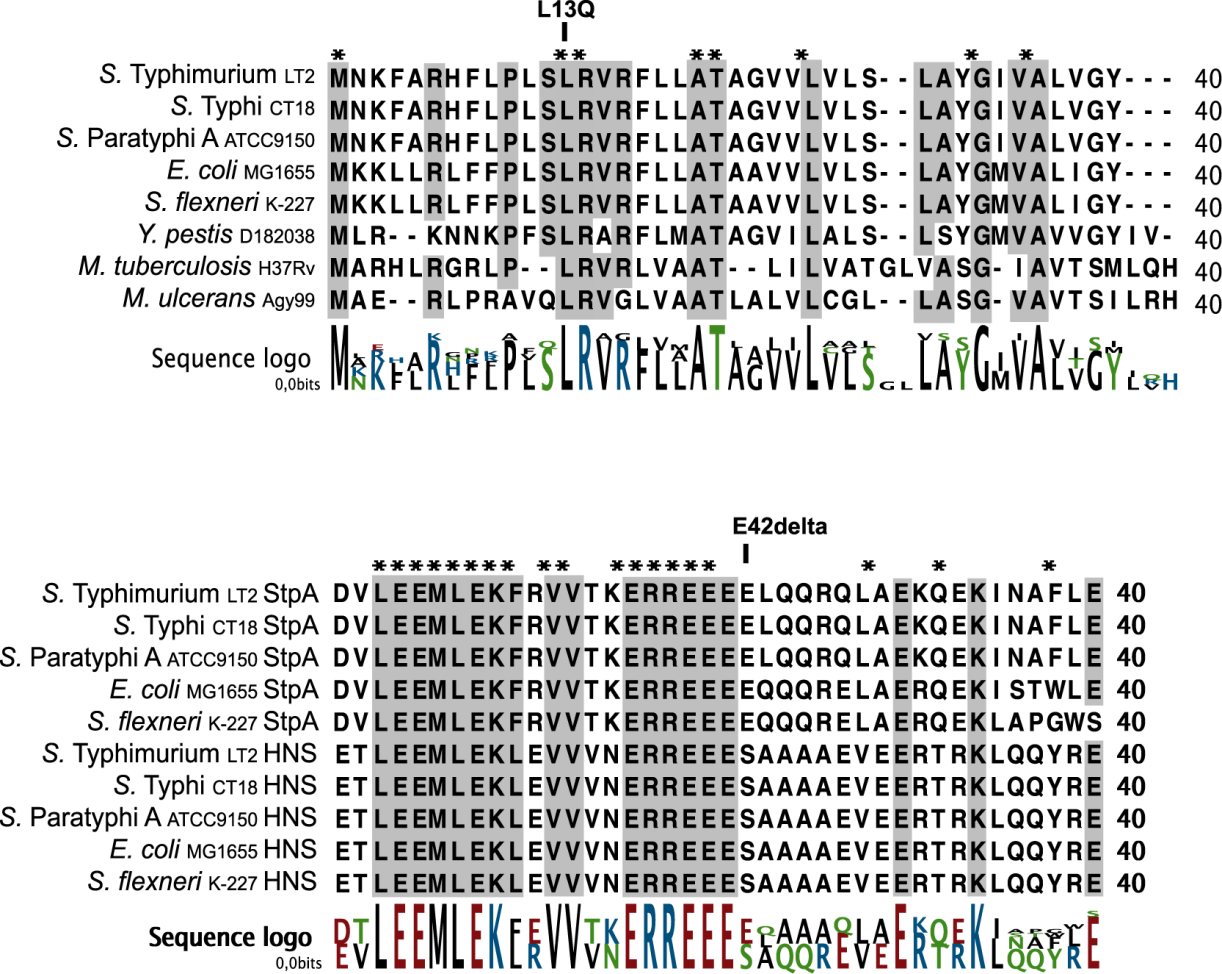
Pathoadaptive *phoQ* (L13Q) and *stpA* (E42delta) mutations are not found in previously sequenced strains. Multiple alignment of PhoQ (top) and StpA and HNS (bottom) proteins from all published Salmonella sequences and other organisms as indicated.

To ascertain whether any other genome sequences contained similar variant alleles of PhoQ and StpA, we conducted BLASTp searches and performed multiple alignments on protein sequences from other *Salmonellae* and related *Enterobacteriaceae* using the variant alleles (Fig. 2). No other Salmonella sequenced to date contained either variant. Furthermore, the L13 residue was conserved in *Salmonella*, *E. coli,Yersinia pestis* and *Mycobacterium* PhoQ homologs. Similarly, the E42 residue of StpA is part of a stretch of four glutamic acid residues that is conserved in the homologues found in *Salmonella*, *E. coli* and *Shigella flexneri* (Fig. 2). However, the StpA homologue H-NS shares sequence identity to StpA in this region but only contains three glutamic acid residues (like our E42delta mutant). According to the domain structure of StpA and H-NS the glutamic acid region is located in the N-terminal domain of the protein and in the region involved in dimerization of H-NS and StpA [27]. Dimers of StpA/H-NS are much more stable than oligomers which are rapidly degraded by Lon protease. Thus the deletion of amino acid E42 could result in increased stability of the StpA protein [14].

### Reconstruction of the Mutants and Growth Rates in Mice

The *stpA* (E42delta) mutation was found in 60% of the population by whole genome sequencing and the original population DA5810 had a competitive index of 57 and a 40% increase in relative fitness (W) (Fig. 1ab). When the mutation was moved to a wild type background the *stpA* (E42delta) mutant had a competitive index of 5 and a 17% increase in relative fitness (Fig. 1a–b). Likewise, the *phoQ* (L13Q) mutation was found in 57% of the population, and the original population DA5816 had a competitive index of 25 and a 32% increase in relative fitness (Fig. 1a–b). When the mutation was moved to a wild type background the *phoQ* (L13Q) mutant had a competitive index of 3 and a 11% increase in relative fitness (Fig. 1a–b). From these results we conclude that the *stpA* (E42delta) and *phoQ* (L13Q) are only partly responsible for the increased virulence seen in the evolved population in mice.

### Regulation of Virulence-gene Expression

PhoQ and StpA are known regulators of virulence genes and both have been shown to affect expression of the RpoS regulon [24], [28]. In turn, RpoS regulates many genes required for virulence including the *spvABCD* gene operon essential for survival within macrophages [29]. To test whether the mutations in StpA and a PhoQ were affecting protein function positively or negatively, we tested how the reconstructed mutations affected expression of the RpoS regulated *katE* and the PhoQ regulated *mgtA* genes known to be regulated by StpA and PhoQ respectively [24], [30]. We also tested whether the mutations affected expression of the RpoS regulated *spvABCD* virulence gene operon (*spv* for *Salmonella* plasmid virulence) required for survival in macrophages.

**Figure 3:**
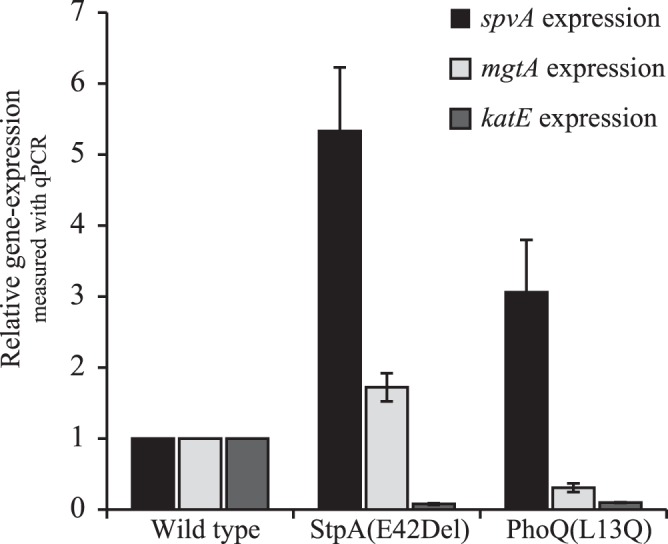
Expression levels of *spvA* (black bars), *mgtA* (light grey bars) and *rpoS* (dark grey bars) in the StpA(E42Del) and the PhoQ(L13Q) mutant relative to wild type levels. All expression levels are relative to the *dnaE* mRNA levels in each RNA sample. Error-bars are SEM.

Quantitative real-time PCR showed that both the reconstructed PhoQ(L13Q) and the StpA(E42Del) mutations resulted in a 10-fold decrease in expression of *katE* (Fig. 3). StpA is known to repress expression of the RpoS regulon (including *katE*) during exponential growth [24]. We observed a 10-fold decrease in *katE* expression in the StpA(E42Del) mutant indicating that the mutation enhances StpA activity. PhoQ stabilizes RpoS levels through increased expression of IraD, which in turn sequesters RssB protein thus preventing RssB from binding to RpoS and delivering it for ClpXP proteolysis [28]. We observed a 10-fold decrease in *katE* expression in the PhoQ mutant, indicating that the PhoQ(L13Q) mutation is affecting PhoQ function negatively. In addition, we tested how the PhoQ(L13Q) mutation affected expression of the *mgtA* gene, that is known to be positively regulated by PhoQ. The PhoQ(L13Q) mutation resulted in a 3-fold decrease in *mgtA* expression levels (Fig. 3) suggesting that the PhoQ(L13Q) mutation is affecting PhoQ function negatively. The StpA(E42Del) mutation did not affect *mgtA* expression as predicted (Fig. 3).

Importantly, both the StpA(E42Del) mutation and the PhoQ(L13Q) mutation resulted in an increased expression of *spvA,* 5- and 3-fold respectively (Fig. 3). Both StpA and PhoQ have been shown to affect expression of the RpoS regulon and RpoS is required for *spv* expression during stress conditions [31]. However, neither the *phoQ* nor the *stpA* mutation increased expression of the RpoS regulon suggesting that the increased *spvA* expression is mediated through another mechanism than increased levels of RpoS.

### Flagellum Inversion in Adapted Strains

**Figure 4:**
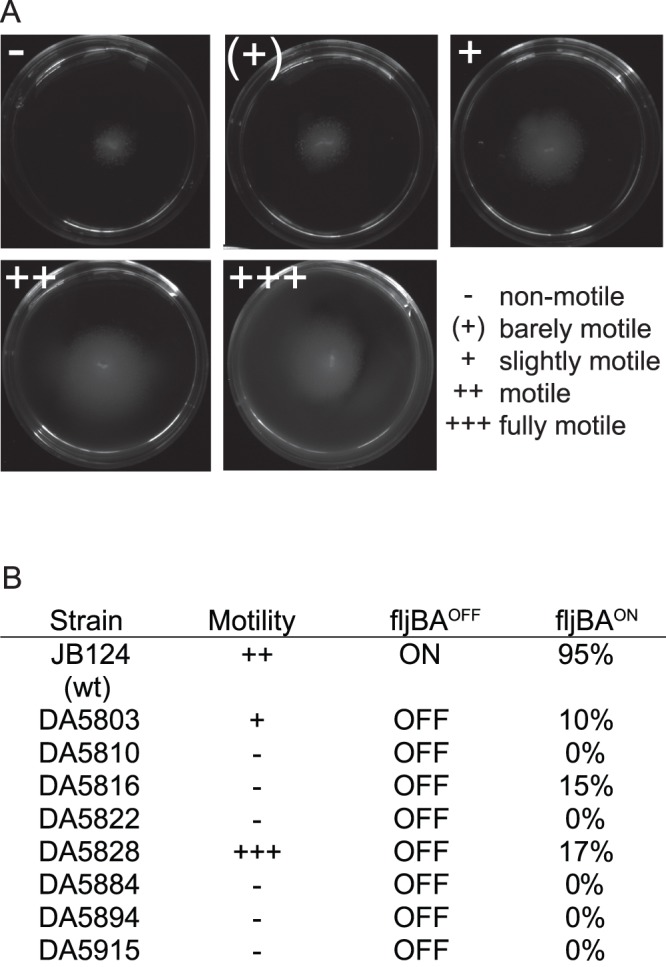
Motility testing on motility agar. A) Illustrating how motility is scored on motility agar. Motility is scored on a 5 step scale where (−) is non-motile and+++is fully motile. B) Motility and phase variation of wild type and evolved Salmonella typhimurium strains. FljBA^ON^ cells expressing *fljB* type of flagellum and FljBA^OFF^ cells expressing *fliC* type of flagellum. Percentage indicates the fraction of cells (based on whole-genome sequencing) in mouse-adapted population that were in FljBA^ON^ state.

The *fljB* and *fliC* genes of *S. typhimurium* are divergently expressed, where a 931 bp invertible cassette that contains a single promoter controls the expression from both *fljB* and *fliC* loci in the following manner: when the *hin*-invertable element is located so that the *fljBA* promoter is in the ON-orientation this results in the expression of both the flagellar protein FljB and the FljA repressor protein. FljA represses expression of *fliC* loci post-transcriptionally, which results in a FljB type of flagellum [10], [24]. When the *hin*-invertable element is located so that the *fljBA* promoter is in the OFF-orientation, this represses expression of both the *fljB* and *fljA* genes. Since FljA no longer represses expression of *fliC*, this orientation of *hin* results in a FliC type of flagellum [10]. Strain JB124 had the *hin* region in the *fljB* ^ON^ orientation and thus expressed the *fljB* type flagellum whereas all of the *in vivo* evolved strains had switched *hin* orientation and was in a *fljB* ^OFF^ state, and were with one exception non-motile (Fig. 4ab).

### Expression of FliC Flagellum Increases Competitive Ability in Mice

Previous results show that *S. typhimurium* mutants expressing FliC type of flagellum are more virulent in mice, at least when administered orally or intravenously [32]. In view of these results, it is possible that in addition to the *phoQ* and *stpA* mutations the switch from *fljB* ^ON^ to *fljB* ^OFF^ contributed to the increased competitive ability of the evolved strains. Competition experiments (with bacteria injected intraperitoneally) with mutants genetically locked in either position (expressing *fliC* or *fljB* exclusively) in wild type background showed that with the flagellum in the *fliC* orientation (*fljBA* ^OFF^, DA21179) the competition index was 5 and a 16% increase in relative fitness as compared to the wild type strain with the flagellum in *fljB* orientation (*fljBA* ^ON^, DA21185) (Fig. 1a–b). Furthermore, combination of the *stpA*(E42delta) with a *fliC* flagellum (*fljBA* ^OFF^, DA18933) resulted in a competitive index of 19 and a 29% increase in relative fitness, to be compared with the original mouse-adapted population that had a competitive index of 57 and a 40% increase in relative fitness (Fig. 1a–b). Combination of the *phoQ* (L13Q) mutation with a *fliC* flagellum (*fljBA* ^OFF^, DA18939) resulted in a competitive index of 4.4 and a 15% increase in relative fitness, to be compared with the original mouse-adapted population with a competitive index of 25 and a 32% increase in relative fitness (Fig. 1a–b). From these results, we conclude that the major part of the increased growth rate in mice seen in the original mouse-adapted population of DA5810 is due to the *stpA* (E42delta) mutation in combination with the *hin*-inversion (29- and 40% increase in relative fitness respectively). However, for the mouse-adapted population of DA5816, the *phoQ* (L13Q) and *hin*-inversion mutations accounted only for a minor part of the increased growth rate in mice (15 and 32% increase in relative fitness respectively). Since several low abundance mutations were found in the latter population, it is possible that these mutations also contribute to the faster growth, thus providing a possible explanation for the difference in virulence between the parental population and the reconstructed strain. It is also possible that several beneficial mutations are competing within the population, resulting in clonal interference and the lack of additive effects on fitness when combining the different mutations. Also, the increased virulence of lineages DA5828, DA5884, DA5894 and DA5915, can be attributed to the *hin*-inversion seen in all the adapted lineages, since this inversion alone should give a competitive index of ∼5 and a 16% increase in relative fitness (Fig. 1a–b).

### Evolved Strains have Partly or Fully Lost their Motility on Motility Agar Plates

To test whether the motility of cells expressing *fliC* type of flagellum rather than *fljB* type flagellum was altered, we performed motility testing on motility agar plates as described previously [21]. All 8 evolved strains were tested, and interestingly, 7/8 strains expressing the *fliC* type of flagellum were non-motile on motility agar plates (Fig. 3a–b), implying that absence of motility increases bacterial growth in mice. The previous finding that *Salmonella* mutants that lack flagella grow significantly faster in BALB/c mice than the flagellated strains supports this hypothesis [7].

## Discussion

Here we show that mutations in global regulators of virulence (*stpA* and *phoQ*) and mutations affecting the flagellum composition increase bacterial growth rates in mice and confer a pathoadaptive phenotype. StpA and PhoQ proteins govern master regulatory systems. PhoQ is the primary sensor kinase involved in regulation of a broad suite of virulence factors, including SPI-1, SPI-2 and various lipid A modifying genes [33]. The effect of the L13Q substitution on PhoQ activity is likely to be subtle, as mutations to PhoQ that result in constitutive activation or complete ablation also attenuate virulence [34]. While the sensor kinase domain at the C-terminus and the Mg2^+^ binding periplasmic loop are well-characterized [25], [30], [35], [36], little is known about the short N-terminal region (including amino acid 13) that precedes the first trans-membrane domain and is predicted to lie in the cytoplasm. This is the first evidence implicating the N-terminal trans-membrane region of PhoQ in any function. Increases in gene expression of members of the PhoP/Q regulon have been observed in hypervirulent strains following animal passage [37], although the transient increase in virulence in those strains suggest different mechanism(s) contributing to the infectivity of those strains.

StpA is a H-NS homologue that in *S. typhimurium* shares 52% amino acid identity with H-NS. Although no obvious phenotype of StpA inactivation could be detected in *E. coli* [38] a recent study showed that inactivation of StpA resulted in altered expression of 5% of the *S. typhimurium* genome [24]. Interestingly, StpA was shown to be a master regulator governing the stationary phase response along with RpoS. LT2 strains of Salmonella have a known defect in the RpoS response which is conferred by a mutation in the start codon resulting in the sub-optimal UUG start. This defect causes a significant decrease in virulence in mice relative to 14028s or SL1344 strains [39], [40]. In spite of the potential advantage of reversion to an AUG start codon, we did not observe any revertants of the *rpoS* gene to the parental allele in any of our evolved strains. However, both StpA and PhoQ have been shown to affect expression of the RpoS regulon in *S.typhimurium*, suggesting that suboptimal expression of the RpoS regulon could be a problem during infection.

RpoS is a stationary phase specific sigma factor that functions as a global regulator of gene expression under various stress conditions. During infection RpoS regulates a number of known virulence factors and is essential for *Salmonella* virulence in mice. For example, RpoS regulates the *spv* (*Salmonella* plasmid virulence) operon required for survival within macrophages. The *spv* locus is required for bacterial virulence in mice, as demonstrated by a several logs increase in LD_50_ dose in an *spv* defective mutant [41]. The *spvABCD* operon is expressed from a single promoter that is positively regulated by both RpoS and the SpvR regulatory protein [42], [43]. Surprisingly, both the StpA(E42Del) and the PhoQ(L13Q) mutations down-regulated expression of the RpoS regulon instead of resulting in an increase in RpoS regulated gene-expression. However, both mutations did increase expression of the *spv* virulence-gene operon specifically in an RpoS independent manner. H-NS, a SptA homologue, has been shown to affect *spv* expression independently of RpoS^44^. H-NS prevents recognition of the *spvR* promoter by a sigma70 containing RNA polymerase and by doing so, couples the expression of the *spv* operon to the cellular levels of sigmaS [44]. StpA is similar to H-NS in that it is able to bind and form a rigid filament along DNA [45]. Even though the DNA binding patterns of StpA and H-NS are similar in *E.coli* [46], major differences in effects on gene regulation are seen in *S.typhimurium* [24]. It is possible that stabilization of the StpA dimer by the StpA(E42Del) mutation allows StpA to increase *spv*-gene expression by changing the DNA curvature at the *spv* locus thus allowing expression of the *spvR* inducer that is normally repressed by H-NS. Increased levels of *spvR* would then lead to increased expression of the *spvABCD* operon as SpvR is essential for activation of the *spvA* promoter and is known to be able to induce RpoS-independent expression from the *spvA* promoter [31]. In conclusion, we have identified two new mutations that both increase expression of the *spv* virulence genes required for survival in macrophages, indicating that *spv* gene expression is limiting *Salmonella typhimurium* during infection and that any mutation that allows increased expression of these genes will increase cell survival. As *Salmonella typhiumurium* LT2 has lower levels of RpoS due to its suboptimal start codon and RpoS is required for full expression of the *spv* operon during stress, one could hypothesize that both the StpA(E42Del) and PhoQ(L13Q) mutations are compensating for the lack of RpoS during infection.

The biological significance of flagellar phase-variation for *Salmonella typhimurium* has been studied in murine infection models. Non-flagellated mutants of *Salmonella typhimurium* retain their virulence and their ability to infect BALB/c mice [46]. However, Salmonella mutants locked in the *fljB* ^ON^ position are completely attenuated in a murine model whereas mutant locked in the *fljB* ^OFF^ position retain their virulence [32]. These experiments were performed as single infections and can therefore not be directly compared with our results obtained from competitions. Our data shows that the *fljB* ^OFF^ locked mutants have a 5-fold increase in competitive indexes in mice as compared to the *fljB* ^ON^ state. Thus, for four of the eight sequenced lineages (DA5822, 5884, 5894, and 5915) the switch from *fljB* ^ON^ to *fljB* ^OFF^ during evolution in mice can explain the increase in competitive ability. As flagellum switching is a frequent event, occurring at a frequency about 10^−3^ to 10^−5^ per cell per generation^21^, it is expected that the majority of cells in all lineages would be in the *fljB* ^OFF^ state. In contrast, the *stpA* and *phoQ* point mutations are much rarer and it will therefore take a longer time for these mutations to appear and become fixed in the population (note that these mutations were only fixed to about 60%).

The contributions to bacterial growth rates by these mutations alone were fairly moderate (11- and 17% increases in relative fitness). However, when two of the mutations (*stpA* and *fljBA* ^OFF^) were combined in the same background this resulted in a synergistic increase in growth. In contrast, combination of the *phoQ* mutation with *fljBA* ^OFF^ resulted in a slightly antagonistic effect on growth, suggesting that the contribution of individual mutations to the fitness of a population is not simply additive. In addition, among the eight whole-genome sequenced lineages several other mutations were found. Thus, it is likely that the mouse-adapted populations contain, apart from the three identified mutations, other low-abundance variants that also contribute to the increased fitness in mice. Interestingly, two of these low abundance mutations were found in two separate lineages (Table 2), which might suggest a strong epistasis between the two mutations. Another possibility is that the strains were contaminated at some point during the evolution experiment. If this was the case, it must have been early during serial passage since both lineages have additional unshared mutations (Table 2).

As our previous report suggested [10], pathoadaptation of these isolates appears to have occurred sequentially, with the high-frequency *hin* inversion event governing flagellum biosynthesis and motility occurring early and in all of the adapted lineages, while lower-frequency point mutations swept the populations later if at all. Our observations are consistent with earlier reports of the requirement for multiple mouse passages of *Burkholderia pseudomallei* to obtain a highly pathogenic isolate [47] and with the rapid purification of virulent isolates from a less-virulent population by single rounds of animal passage [48]. The emergence of pathoadaptive mutations in nature is restricted by the mutation rate of the pathogen and its effective population size at multiple points in the infection cycle, especially the genetic bottlenecks associated with transmission between individuals and even within hosts [49], [50]. Our results show that the pathogen’s own evolved regulatory network itself provides an additional constraint on *in vivo* evolution that must be re-tuned to accommodate changes in host environments.

## Supporting Information

Table S1 **Genome Sequencing Statistics by Strain.**
(DOCX)

Table S2 **Sequence differences between strain JB124 and the published sequence of** ***S. typhimurium*** **LT2 (Genbank ID NC003197).**
(XLSX)

Table S3 **Unique Mutations identified in JB124 relative to LT2.**
(DOCX)

Table S4 **Primers used.**
(DOCX)
