# Supplementary material for: Pathoadaptive Mutations in Salmonella enterica Isolated after Serial Passage in Mice
Source: PLoS One. 2013 Jul 25;8(7):e70147. doi: 10.1371/journal.pone.0070147 (PMC3723669; doi:10.1371/journal.pone.0070147)
Supplement: Table S1 — Genome Sequencing Statistics by Strain. (DOCX) [file pone.0070147.s001.docx]

**Table S1**. Genome Sequencing Statistics by Strain.

|  | **De novo Assembly Statistics** | | | | | **Mapping to LT2 Chromosome and Plasmid^1^** | | | |
| --- | --- | --- | --- | --- | --- | --- | --- | --- | --- |
| **Strain** | **# Aligned Reads (% total)** | **Read Length**  **(Avg)** | **Median Depth of coverage** | **# contigs >500 bp (N50, kb)** | **% Bases Q40+** | **% coverage of LT2 Chromosome** | **Depth of LT2 chromosome coverage** | **% coverage pSLT1** | **Depth of pSLT1 coverage** |
| **JB124** | 567497 (99.38) | 338 | 38 | 54 (178) | 99.97 | 99.42 | 38x | 100.00 | 79x |
| **DA5803** | 455688 (99.43) | 348 | 31 | 50 (198) | 99.97 | 99.38 | 31x | 100.00 | 67x |
| **DA5810** | 511424 (99.33) | 321 | 32 | 61 (178) | 99.96 | 99.34 | 33x | 100.00 | 69x |
| **DA5816** | 349444 (99.61) | 351 | 24 | 59 (167) | 99.90 | 99.47 | 24x | 100.00 | 53x |
| **DA5822** | 532804 (99.55) | 384 | 31 | 47 (192) | 99.97 | 99.38 | 40x | 100.00 | 86x |
| **DA5828** | 541557 (99.48) | 376 | 40 | 57 (149) | 99.98 | 99.40 | 40x | 100.00 | 86x |
| **DA5884** | 407735 (99.59) | 366 | 29 | 56 (178) | 99.92 | 99.51 | 29x | 100.00 | 63x |
| **DA5894** | 565039 (99.48) | 376 | 42 | 53 (192) | 99.98 | 99.38 | 42x | 100.00 | 95x |
| **DA5915** | 464721 (98.93) | 379 | 35 | 45 (223) | 99.96 | 99.36 | 35x | 100.00 | 78x |

^1^References used were NC_003197.1 and NC_003277
