# Supplementary material for: Pathoadaptive Mutations in Salmonella enterica Isolated after Serial Passage in Mice
Source: PLoS One. 2013 Jul 25;8(7):e70147. doi: 10.1371/journal.pone.0070147 (PMC3723669; doi:10.1371/journal.pone.0070147)
Supplement: Table S3 — Unique Mutations identified in JB124 relative to LT2. (DOCX) [file pone.0070147.s003.docx]

**Table S3.** Unique Mutations identified in JB124 relative to LT2.

| **Coordinates (LT2 genome)** | **Reference Sequence** | **JB124 (454)** | **Effect on Amino acid sequence** | **Gene, annotation** | **LT2 Locus Tag** | **Other Salmonella Strains^1^** |
| --- | --- | --- | --- | --- | --- | --- |
| 290717 | C | A | - | 16S rRNA, *rrsH* | STM0249 | C |
| 453938 | C | T | Synonymous | Branched-chain amino acid transporter, *brnQ* | STM0399 | C |
| 608856 | G | C | G61A | Minor fimbrial subunit, *fimH* | STM0547 | G^2^ |
| 691691 | C | T | G29D | C4-dicarboxylate transporter, *dcuC* | STM0627 | C |
| 1795046 | G | A | W234Stop | Exoribonuclease II, *rnb* | STM1702 | G |
| 1841404 | G | A |  | Intergenic |  | G |
| 1849648 | A | C | V102G | Response regulator of RpoS, *hnr* | STM1753 | A |
| 2020200 | G | A | P271L | Flagellar motor protein, *motA* | STM1923 | G |
| 2286207 | A | T | V93E | Galactose/methyl galactoside transporter ATP-binding protein, *mglA* | STM2189 | A |
| 3673617 | A | C | L96R | 4-alpha-glucanotransferase, *malQ* | STM3513 | A |
| 3674904 | 37 bp | - | 37bp deletion | Maltodextrin phosphorylase, *malP* | STM3514 | 37 bp |
| 3819803 | T | C | Synonymous | Putative regulatory protein | STM3633 | T |
| 4122923-4124126 | 1202 bp | - | Deletion of 5’ region of *gppA* and 3’ region of *rhlB* | *gppA –rhlB* | STM3913-STM3914 | 1202 bp |

^1^Identified by BLASTn searches of Genbank non-redundant database limited to “Salmonella”

^2^G61A allele is present in some LT2-derived strains but not in the published sequence [42].
