# Supplementary material for: Pathoadaptive Mutations in Salmonella enterica Isolated after Serial Passage in Mice
Source: PLoS One. 2013 Jul 25;8(7):e70147. doi: 10.1371/journal.pone.0070147 (PMC3723669; doi:10.1371/journal.pone.0070147)
Supplement: Table S4 — Primers used. (DOCX) [file pone.0070147.s004.docx]

**Table S4.** Primers used for DNA sequencing, PCR and qPCR.

Name Sequence

STM2804_cat_insF GCACAGTAAAGCGCTGCGTAAGGCTGGCGTG

AATCAAGCTtgtaggctggagctgcttc

STM2804_cat_insR CTGAAATGGTGACTTACCCGCCATCCTGCC

AGCGCATCTAcatatgaatatcctcctta

STM1236_cat_insF ATTAACACCGTTATCCGTTAATGCGGCC

AGCTTCGACTGCCtgtaggctggagctgcttc

STM1236_cat_insR GTGCGCGCAGATAGCCTTTTCATCCGCAG

CCTGCGCTTTCTcatatgaatatcctcctta

phoP_F CATTATGGAAACGCTTATCCG

phoQ_R TCCCATTTGGCGAGGGTATA

stpA_F ATGAATTTGATGTTACAGAACT

stpA_R GCATTGCTGAATCCATGGCA

katEF(rt) GCGGTATTCCACGCAGTTAT

katER(rt) CAGTGGAAACGGACAAAGGT

spvAF(rt) TATTGTCCGTCAGACCCGTA

spvAR(rt) ACATGCAGCGGATATACTGC

mgtAF(rt) AATGGAGCAGGAGACTCTCT

mgtAR(rt) GCGATAACATACCCACAGATG

dnaEF(rt) GAACCACGTTTCGTTCACCT

dnaER(rt) GACCGCAGAGGTTGGTAAAA
